# Body Metrics and the Gut Microbiome in Response to Macronutrient Limitation in the Zebrafish *Danio rerio*

**DOI:** 10.1016/j.cdnut.2023.100065

**Published:** 2023-03-09

**Authors:** George B.H. Green, Michael B. Williams, Sophie B. Chehade, Jonathan T. Flowers, Casey D. Morrow, Addison L. Lawrence, Asim K. Bej, Stephen A. Watts

**Affiliations:** 1 Department of Biology, The University of Alabama at Birmingham, Birmingham, AL, United States; 2 Department of Cell, Developmental and Integrative Biology, The University of Alabama at Birmingham, Birmingham, AL, United States; 3 Texas A&M AgriLife Extension Agriculture and Life Sciences, College Station, TX, United States; 4 J. Frank Barefield, Jr. Department of Criminal Justice, The University of Alabama at Birmingham, Birmingham, AL, United States

**Keywords:** diet, homeostasis, nutrition, PICRUSt2, 16S rRNA, QIIME2

## Abstract

**Background:**

Healthy and predictable physiologic homeostasis is paramount in animal models for biomedical research. Proper macronutrient intake is an essential and controllable environmental factor for maintaining animal health and promoting experimental reproducibility.

**Objective and Methods:**

Evaluate reductions in dietary macronutrient composition on body weight metrics, composition, and gut microbiome in Danio rerio.

**Methods:**

D. rerio were fed reference diets deficient in either protein or lipid content for 14 weeks.

**Results:**

Diets of reduced-protein or reduced-fat resulted in lower weight gain than the standard reference diet in male and female *D. rerio*. Females fed the reduced-protein diet had increased total body lipid, suggesting increased adiposity compared with females fed the standard reference diet. In contrast, females fed the reduced-fat diet had decreased total body lipid compared with females fed the standard reference diet. The microbial community in male and female *D. rerio* fed the standard reference diet displayed high abundances of *Aeromonas*, Rhodobacteraceae, and *Vibrio*. In contrast, *Vibrio* spp. were dominant in male and female *D. rerio* fed a reduced-protein diet, whereas *Pseudomonas* displayed heightened abundance when fed the reduced-fat diet. Predicted functional metagenomics of microbial communities (PICRUSt2) revealed a 3- to 4-fold increase in the KEGG (Kyoto Encyclopedia of Genes and Genomes) functional category of steroid hormone biosynthesis in both male and female *D. rerio* fed a reduced-protein diet. In contrast, an upregulation of secondary bile acid biosynthesis and synthesis and degradation of ketone bodies was concomitant with a downregulation in steroid hormone biosynthesis in females fed a reduced-fat diet.

**Conclusions:**

These study outcomes provide insight into future investigations to understand nutrient requirements to optimize growth, reproductive, and health demographics to microbial populations and metabolism in the *D. rerio* gut ecosystem. These evaluations are critical in understanding the maintenance of steady-state physiologic and metabolic homeostasis in *D*. *rerio*. *Curr Dev Nutr* 20xx;x:xx.

## Introduction

The initial usage of a *Danio reri*o model in applied biomedical research originated from the advantages in short-generation time, high-fecundity levels, and transparent larval development allowing in vivo observation [1]. In addition, *D. rerio* has been used in nutrition research because of the physiologic and anatomic similarities of their digestive system to mammals [2,3]. Deep sequencing of *D. rerio* revealed 70% of human genes have a minimum of 1 *D. rerio* ortholog, including a highly conserved genomic region of intestinal development and physiology [3,4]. The mammalian gastrointestinal tract comprises 5 segments: the stomach, duodenum, jejunum, ileum, and colon. In comparison, *D. rerio* is divided into 3 distinct segments: the anterior bulb, middle intestine, and posterior intestine. *D. rerio* lacks a true stomach, with no acidification occurring in digestion; however, after transcriptomic investigation, conserved transcriptional domains in *D. rerio* and mammalian digestive physiology were revealed, with correlations between the anterior bulb, duodenum regions, anterior, jejunum regions, middle, ileum regions, and middle-to-posterior, colon-comparative regions [5]. *D. rerio* gut segments share characteristics involved in mammalian digestion, with the anterior intestinal bulb recovering bile salts, the middle intestine absorbs lipids and proteins, and the posterior intestine absorbs water and ions [3]. This homology between human and *D. rerio* provides an opportunity to study the linkage of diet, digestion, and the gut microbial communities in the maintenance of host health, metabolism, or the potential to manifest disease because of dysbiosis [[5], [6], [7], [8]].

In *D. rerio*, the gut microbiome colonization cycle initiates from microorganisms in their environment [6,9]. In contrast, mammals acquire their initial microbiota from birth, or before birth from the mother’s womb and reach their adult microbiome composition approximately at the age of 3-y-old [1,10]. *D. rerio* has been previously documented to be colonized throughout all stages of life with members of the phylum Proteobacteria, Firmicutes, and Fusobacteria, which is common amongst teleost fish [6]. Despite differences in the colonization process, several gene regulatory pathways of the gut microbiota in *D. rerio* reveal similarities to mice and humans, particularly in nutrient and xenobiotic metabolism, epithelial cell turnover processes, and innate immune responses [11]. Similar to mammalian models, *D. rerio* microbiota can impede metabolic health because gut dysbiosis is connected to imbalances in host metabolism, intestinal and extraintestinal disorders, pathogenesis, and progression of disease [[12], [13], [14]].

In research laboratories, *D. rerio* is typically provided 1 of several commercially available diets, all of which are currently proprietary in ingredient composition and cannot be used as reference diets [2,15,16]. Dennis-Cornelius et al. (2022) [17], Williams et al. [15], and Karga and Mandal (2017) [18] have reported growth and body composition outcomes that were the result of the use of open-formulation defined diets in *D. rerio*. In this study, we used a reference diet to validate the link between diet, growth and reproductive outcomes, and the gut microbiome.

Similar to most species, *D. rerio* most likely has specific requirements for the dietary intake of organic macronutrients when held under standard husbandry conditions and fed reference diets. Diets that do not satisfy macronutrient requirements or have an imbalance of macronutrient content (e.g., lower protein-energy ratio, specific amino acid, or fatty acid deficiencies, etc.) can result in disease states and introduce variability in study outcomes [19]. In *D. rerio*, dietary macronutrient quantity and quality, particularly in proteins and lipids, have been shown to affect growth outcomes [20,21]. A 2016 study in which a 2-mo-old *D. rerio* was fed diets of variable protein content revealed that growth was positively correlated with dietary protein content up to a maximal level of 44.8% dietary protein [22]. Diets of lower protein content (which by formulation necessitates a lower protein-energy ratio) resulted in an increased intake of dry matter and EI. *D. rerio* on the lower protein diets also had decreased carcass protein and moisture, suggesting increased adiposity. This increased adiposity and increased consumption in the lower protein diets match predictions of the protein leverage hypothesis [23,24]. Total lipid requirements have been estimated by Fowler et al. [25,26]. Compared with proteins and lipids, carbohydrate requirements are estimated to be minimal, but soluble carbohydrates are required for optimal function [9]. Collectively, these macronutrients are necessary to insure adequate health.

Modifying macronutrient ratios alter microbial populations inhabiting the *D. rerio* gut ecosystem, and additionally the surrounding microbes in the environment [27]. Optimized ratios of proteins, carbohydrates, and lipids are key for development, reproduction, and metabolic health, but altering diet compositions reveals distinct changes in microbial populations, and potential inflammatory phenotypic changes in *D. rerio*, and other models, including mice and humans [28,29]. For example, in *D. rerio,* induction of high-fat-diet results in higher abundance of Bacteroides species, which leads to an overexpression of the inflammatory marker NF-κβ, and genes relating to antimicrobial metabolism, resulting in intestinal damage [30]. Evaluating macronutrients in *D. rerio* confirms the importance of having a reference diet, providing an opportunity to study the linkage between diet, digestion, and the microbial role in metabolic regulation.

In this study, a standard reference (SR) diet has been compared with a diet that restricts dietary protein content (while increasing carbohydrate content) and to a diet that restricts total dietary lipids. This study provides insight into the effects of defined macronutrient levels on body metrics, fecundity, microbial composition, and their associated functional metagenomic profiles in *D. rerio*.

## Methods

### Experimental housing and husbandry

All procedures for vertebrate animal study were approved by the UAB IACUC (Institutional Animal Care and Use Committee) and adhere to standard *D. rerio* husbandry requirements for housing and euthanasia under the permit IACUC-20656, 29/10/2014, S.A. Watts. *D. rerio* embryos (AB strain) were randomly collected from a mass spawning of males and females. Embryos were transferred to Petri dishes (*n* = 50 per dish) and incubated at 28.5 °C until 5 d postfertilization (dpf). At 5 dpf, hatched larvae were polyculture in 6-L static tanks (*n* = 100 larvae per tank) with the rotifer *Branchionus plicatilis* L-type (Reed Mariculture) at a salinity of 5 ppt, and enriched with a blend of 6 microalgae (RotiGrow Plus, Reed Mariculture). At 11-dpf all tanks were placed on a recirculating aquaculture system (ZS560 Standalone System, Aquaneering) and were fed stage-1 *Artemia nauplii* until 28-dpf. At 28-dpf, all 6-L tanks were combined, and fish were randomly distributed into 2.8-L tanks with 14 fishes per tank. Each tank was then randomly assigned a dietary treatment (10 tanks per treatment) and the feeding trial was initiated. *D. rerio* were fed for a 16-wk period 1 of 3 diets. To obtain initial weights, a subsample of fish (128) was individually weighed before experiment implementation (initial wet weight = 53 mg). For the first 2 wk of the trial, fish receiving powdered feeds were provided a ration of 10% of initial body weight per day. Daily rations were weighed for individual tanks. Rations were adjusted based on weight gain and food conversion ratios every 2 wk. Fish were fed at 08:00 and 16:00 each day (United States Central Time).

All tanks were maintained at 28 °C and 1500 μS/cm conductivity in a commercial recirculating system, with 5.4 L exchanged from each tank per hour. Municipal tap water was passed through mechanical filtration (1-μm sediment filter), an activated carbon filter, reverse osmosis filter, and a cation/anion exchange resin. Synthetic sea salts (Crystal Sea, Marinemix) were added to adjust the conductivity of the system water. Sodium bicarbonate was added as needed to maintain the pH of the system water at 7.4. Total ammonia nitrogen, nitrite, and nitrate were measured colorimetrically once weekly. To help sustain adequate water quality, a water exchange of 10% was performed on the recirculating system daily. The water passes through activated charcoal and UV sterilization on each pass through the system, before re-entering tanks to reduce potential persistent compounds from feed or microbial organisms. Tanks are maintained on the same recirculating system throughout the duration of the experiment; however, to reduce environmental confounding effects from noise, light, vibration, or other unidentified sources, they were cleaned and returned to a randomized new position on the recirculating system every 2 wk. Experimental animals were maintained under a 14-h light/10-h dark cycle with lights turned on at 07:00 local time (United States Central Time). At termination of the feeding trial, all fish were sexed and weighed individually to 0.001g and photographed. All photographs were analyzed with NIS Elements 3.1 software to determine the total body length (measured from tip of snout to the distal end of the caudal peduncle) to 0.01 mm. A subset of from each diet group of female (*n* = 15) and male (*n* = 6–13) *D. rerio* at termination were dried via freeze-drying to determine moisture content, and total lipid for females and males were determined using a protocol of the Folch total lipid extraction optimized for *D. rerio* (Folch et al., 1957) [31]. At the end of the study, fish were killed by rapid submersion in ice-cold water for a minimum of 10 min and left until the opercular motion has ceased. Secondary euthanasia was conducted via decapitation.

### Diet preparation

Each diet was produced from cholesterol, menhaden oil, corn oil, vitamin (MP Biomedicals custom vitamin mixture) and mineral premixes (MP Biomedicals 290284), and alginate binders. The protein sources were fish protein hydrolysate (The Scoular Company, Sopropeche, Cat. no CPSP90) and casein (MP Biomedicals, Cat. no 904798). All ingredients were weighed on an analytic balance (Mettler Toledo New Classic MF Model MS8001S or Model PG503-S Mettler-Toledo, LLC.) and mixed using a Kitchen Aid Professional 600 Orbital Mixer (Kitchen Aid,). The diets were cold extruded into strands to preserve nutrient content using a commercial food processor (Cuisinart) and the strands were air-dried for 24 h on wire trays. The proximate analysis of diets for each of the 3 diet sources was performed by Eurofins. Diets formulated in house included a reference diet of 35% casein, 20% fish protein hydrolysate, and 7.2% added oil (SR diet), a modification of the reference diet resulting in protein content being reduced to 20% dry matter (reduced-protein [RP] diet), and a modification of the reference diet with no added oil (reduced-fat [RF] diet) (Table 1). Because of typical formulation constraints, when dietary protein is reduced, the dry matter content of the diet is offset with carbohydrates at levels that are elevated compared with the reference diet, but less than those eliciting a growth-related response in zebrafish [32]. However, this addition of carbohydrates does reduce the protein:energy and protein:carbohydrate ratios of the RP diet, and this alteration should be noted. For the RF, decreased oils were offset with increased diatomaceous earth, the levels of which are metabolically inert.

**TABLE 1:** Ingredient composition of current formulated standard reference diet (SR), reduced-protein diet (RP), and reduced-fat (RF).

	SR	RP	RF
Casein - low trace metals	35.00	20.00	35.00
Fish protein hydrolysate	20.00	20.00	20.00
Wheat starch	5.65	14.05	5.65
Dextrin type III	1.61	4.00	1.61
Alpha cellulose	1.00	1.00	1.00
Diatomaceous earth	12.57	16.73	19.72
Menhaden fish oil (ARBP) Virginia Prime Gold	2.60	2.60	0.00
Safflower oil	4.55	4.60	0.00
Alginate	2.00	2.00	2.00
Soy lecithin (refined)	4.00	4.00	4.00
Vit Pmx (MP Vit Diet Fortification Mixture)	4.00	4.00	4.00
Mineral Pmx aka premix (AIN 93G)	3.00	3.00	3.00
Canthaxanthin (10%)	2.31	2.31	2.31
Potassium phosphate monobasic	1.15	1.15	1.15
Glucosamine	0.25	0.25	0.25
Betaine	0.15	0.15	0.15
Cholesterol	0.12	0.12	0.12
Ascorbylpalmitate	0.04	0.04	0.04
Total	100.00	100.00	100.00
Calculated protein level (%) as fed	47.35	34.12	47.35
Calculated protein level (%) dry	52.62	37.92	52.62
Cal protein base on amino acids (%) as fed	45.44	32.22	45.44
Calculated protein base on amino acids (%) dry	50.49	35.81	50.49
Calculated lipid level (%) as fed	11.31	11.31	4.88
Calculated lipid level (%) dry	12.57	12.57	5.42
Calculated carbohydrate level (%) as fed	10.12	19.83	10.12
Calculated carbohydrate level (%) dry	11.24	22.03	11.24
Calculated energy level (cal/g) as fed	4149	3790	3541
Calculated energy level (cal/g) dry	4610	4212	3935
Cal energy level using amino acids (cal/g) as fed	4041	3682	3433
Cal energy level using amino acids (cal/g) dry	4490	4092	3814
Protein: energy ratio based on AA dry	112.45	87.51	132.36

All values represent % of total dry matter inclusion except calculated energy which is (cal/g). a. MP Biomedicals 904654: vitamin A acetate (500,000 iu/gm) 1.80000, vitamin D2 (850,000 iu/gm) 0.12500, dl-a-tocopherol acetate 22.00000, ascorbic acid 45.00000, inositol 5.00000, choline chloride 75.00000, menadione 2.25000, p-aminobenzoic acid 5.00000, niacin 4.25000, riboflavin 1.00000, pyridoxine hydrochloride 1.00000, thiamine hydrochloride 1.00000, calcium pantothenate 3.00000, biotin 0.02000, folic acid 0.09000, vitamin b12 0.00135, measures are mg/g. b. AIN 93 mineral mix for envigo (indianapolis, in): sucrose, fine ground 209.496, calcium carbonate 357.0, sodium chloride 74.0, potassium phosphate, monobasic 250.0, potassium citrate, monohydrate 28.0, potassium sulfate 46.6, magnesium oxide 24.3, manganous carbonate 0.63, ferric citrate 6.06, zinc carbonate 1.65, cupric carbonate 0.31, potassium iodate 0.01, sodium selenite 0.0103, chromium potassium sulfate, dodecahydrate 0.275, lithium chloride 0.0174, boric acid 0.0815, sodium fluoride 0.0635, nickel carbonate hydroxide, tetrahydrate 0.0318, ammonium meta-vanadate 0.0066 measures are mg/g.

RF, reduced-fat diet; RP, reduced-protein diet; SR, standard reference diet.

### Egg production and viability

After 16 wk on the treatment diets, 10 female and 10 male fish from each diet were maintained in 2.8-L tanks on the Aquaneering recirculating systems for an additional 4 wk for subsequent breeding analysis. Maintenance conditions and feeding regime continued as described. For each diet, egg production and embryo viability (at 4 and 24 h postfertilization [hpf]) were assessed. Females and males were randomly selected from each tank and paired with *Artemia*-fed males and females, respectively, from the UAB Lab Animal Nutrition Core brood stocks (https://www.uab.edu/norc/cores/animal-models/lab-animal). Breeding pairs (1 male and 1 female) were transferred to 500-mL breeding tanks with a divider separating the pair on the evening before breeding. Dividers were removed when the lights turned on the following morning and allowed a 2-h period for spawning, after which each adult male and female were returned to their respective tanks. Immediately after spawning, eggs/embryos from successful breeding pairs were collected, cleaned, counted, and scored as viable embryos or nonviable eggs. After counting, viable embryos were divided into Petri dishes (*n* = 50) and incubated overnight at 28.5 °C in fresh 0.7 ppt ASW. At 24 hpf, viable embryos were counted again and assessed for normal development based on their morphology. Males from diet treatments were bred once to assess reproductive health, whereas females were bred twice to account for low egg release during a female’s first spawn.

### Statistical modeling and analysis

Data from this study were analyzed using RStudio Statistical Software (R Core Team, 2016, v0.99.896), and graphs were generated with the Statistical Package for Social Science version 2.3 (IBM). All analyses for continuous outcomes were stratified by sex. Terminal body weight, total body length, and body condition index were compared separately by ANOVA. FM was analyzed with ANCOVA, adjusting for body weight as a covariate. Any observed significant differences (*P* < 0.05) were further analyzed with pairwise comparisons among diets using Tukey’s HSD (Honestly Significant Difference) post hoc test. All data were analyzed for normality and equal variances. Any data sets with a nonnormal distribution were log-transformed. For total embryos produced, previous examination of similar data sets has revealed overdispersion with excessive truncated zeroes (nonsuccessful breeding events), indicating that it was well-suited for a hurdle-negative binomial model (Hothorn et al., 2008) [33]. Data for total embryo production were fitted to a hurdle-negative binomial model with the help of the pscl package of the R language [34]. Diet and week were included as predictors in the model and analyzed for main effects on total embryo production. The outcome for embryo viability is a proportion between 0 and 1, with 2 types of zeroes present: truncated (nonsuccessful breeding events) and sampling (0 viable embryos produced). For this reason, a zero-inflated β-regression model is selected as the most appropriate model. The first component of the β-regression model uses logistic regression and the parameter nu (controls the probability that a 0 occurs) to analyze the 0 counts and determine the probability of 0 viable embryos produced. The second component analyzes the positive counts by fitting β-regression to compare the expected proportion of viable embryos and includes the parameters mu (mean) and sigma (variance) (John Dawson, Department of Biostatistics, personal communication). The best-fit model usually includes all 3 parameters and is selected with the help of the gamlss package of the R language [35].

### High-throughput sequencing

At the termination of the 16-wk feeding, 4 male and 4 female *D. rerio* from all 3 dietary regimens had whole guts (stomach and intestine) dissected out and flash-frozen in liquid nitrogen before being transferred to a −80 °C freezer until used for microbiome analysis. The metacommunity DNA samples from *D. rerio* were purified using the Zymo Research kit. High-throughput amplicon sequencing was performed on an Illumina MiSeq using the 250 bp paired-end kits (Illumina, Inc.) and by targeting the V4 hypervariable region of the bacterial 16S rRNA gene [36]. The resultant sequences were demultiplexed and FASTQ formatted [37,38] and then deposited on the National Center for Biotechnology Information Sequence Read Archive under BioProject IDs PRJNA772302 and PRJNA772305 for the RP and RF diet fed *D. rerio*. The *D. rerio* sample groups were labeled for this study as female *D. rerio* fed with the RF diet (*n* = 4), male *D. rerio* fed with the RF diet (*n* = 4), female *D. rerio* fed with the RP diet (*n* = 4), male *D. rerio* fed with the RP diet (*n* = 4), female *D. rerio* fed with the SR diet (*n* = 4), and male *D. rerio* fed with the SR diet (*n* = 4).

### Taxonomic distribution

The taxonomic features of *D. rerio* fed with SR, RP, and RF were determined via QIIME2 (2022.2) [39]. The raw sequence files in FASTQ format were imported into QIIME2 (2022.2) [35] via “qiime tools import” function with the input format cassava 1.8 paired-end demultiplexed fastq format (CasavaOneEightSingleLanePerSampleDirFmt). The now imported qiime2 object was quality checked via the “qiime demux summarize” function. The output file was passed through quality filtering via DADA2 (q2-dada2 denoise-paired) [40]. The denoising results output file was from DADA2 were summarized via the “qiime feature-table summarize” command (Supplemental Data 2). The representative sequences were outputted via the command “q2- feature-table tabulate-seqs.” The DADA2 output statistics were outputted utilizing “qiime metadata tabulate” command. The mafft program plugin (q2-alignment) aligned the outputted amplicon sequence variants (ASVs) [41], and the data file was piped into fasttree2 (q2-phylogeny) to build the phylogeny [42] utilizing the default building method. To generate α-diversity (Simpson [43], Faith’s Phylogenetic Diversity [44], Shannon [45]), and β-diversity metrics [46], unweighted UniFrac [47], Bray–Curtis dissimilarity, was generated via the core-metrics-phylogenetic command via “q2-diversity plugin” [43]. For the core diversity metrics, the samples were rarefied to a minimum of 35692 sequences per sample. The taxonomic ids were then assigned to ASVs via the command q2-feature-classifier [48] plugin utilizing “classify-sklearn” utilizing the silva-138-99-nb-classifier [49]. The Taxonomy assigned via “classify-sklearn” were collapsed into levels and were outputted into table format (tsv format) using “qiime taxa collapse” [35]. The q2-diversity plugin [44] was utilized to generate PERMANOVA statistics via “beta-group-significance,” Adonis statistics using the “adonis” command [43], and permdisp statistics using “permdisp” as the parameter. The linear discriminant analysis (LDA) effect size (version 1.0.8.post1) [50] determined significant differential abundances across male and female *D. rerio* samples. The comparisons were made as SR against RP, and SR against RF diet fed *D. rerio*. Nonparametric Kruskal–Wallis sum-rank test was determined significant differential abundances, at a default setting of *P* = 0.05 [51], and a pairwise Wilcoxon signed-rank test determined differences between classes at a default setting of *P* = 0.05 [52]. The finalized output was used for the LDA analysis at the default threshold [50,53]. The output ASVs of significant effect size were inputted into a divergent plot, to display the LDA effect sizes obtained via statistical analysis of metagenomic profiles utilizing a galaxy hub (https://huttenhower.sph.harvard.edu/lefse/).

### Predicted functional analysis

Phylogenetic Investigation of Communities by Reconstruction of Unobserved States (PiCRUSt2) [54] determined the predicted functional profiles/capacity of the gut microbiota across *D. rerio* samples. The command “picrust2_pipeline.py” outputted hidden-state prediction of genomes, metagenome prediction, sequence placement, pathway-level predication, and Nearest Sequenced Taxon Index values. The descriptions were added to the metagenome predictions via “add_descriptions.py” command, which provides a description of each functional capacity [50]. The KEGG functional profiles were obtained utilizing “custom_map_table” against KEGG profiles descriptions provided in PiCRUSt2. The functional abundances were normalized to the male and female *D. rerio* fed with the SR diet. *D. rerio* samples were divided by the mean of the SR group (SR male samples for male samples and SR female for the female samples). The mean, normalization, and standard were determined and plotted in R (ggplot package) [55].

## Results

### Body composition metrics

**FIGURE 1:**
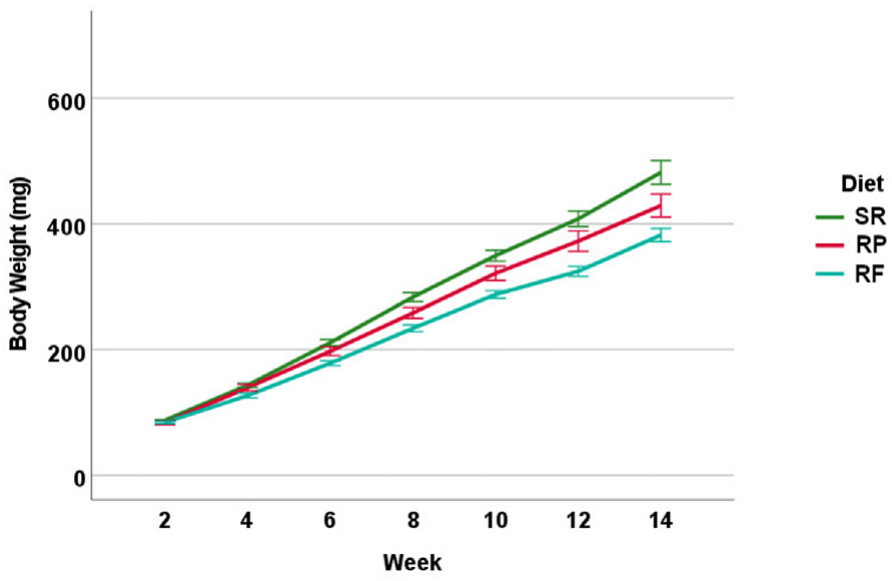
Total body weight average for tanks of fish (mg) for male and female *D. rerio* (combined) measured every 2 wk from wk 2 to wk 14 on the assigned diets (*n* = 10 tanks, 14 fishes per tank for each diet treatment). Sample destinations are as follows: *D. rerio* fed standard reference diet (SR, green line, *n* = 10 tanks), *D. rerio* fed reduced-fat diet (RF, blue line, *n* = 10 tanks), and *D. rerio* fed reduced-protein (RP, red line, *n* = 10 tanks). The line plot was generated via SPSS (version 26).RF, reduced-fat; RP, reduced-protein; SR, standard reference.

**FIGURE 2:**
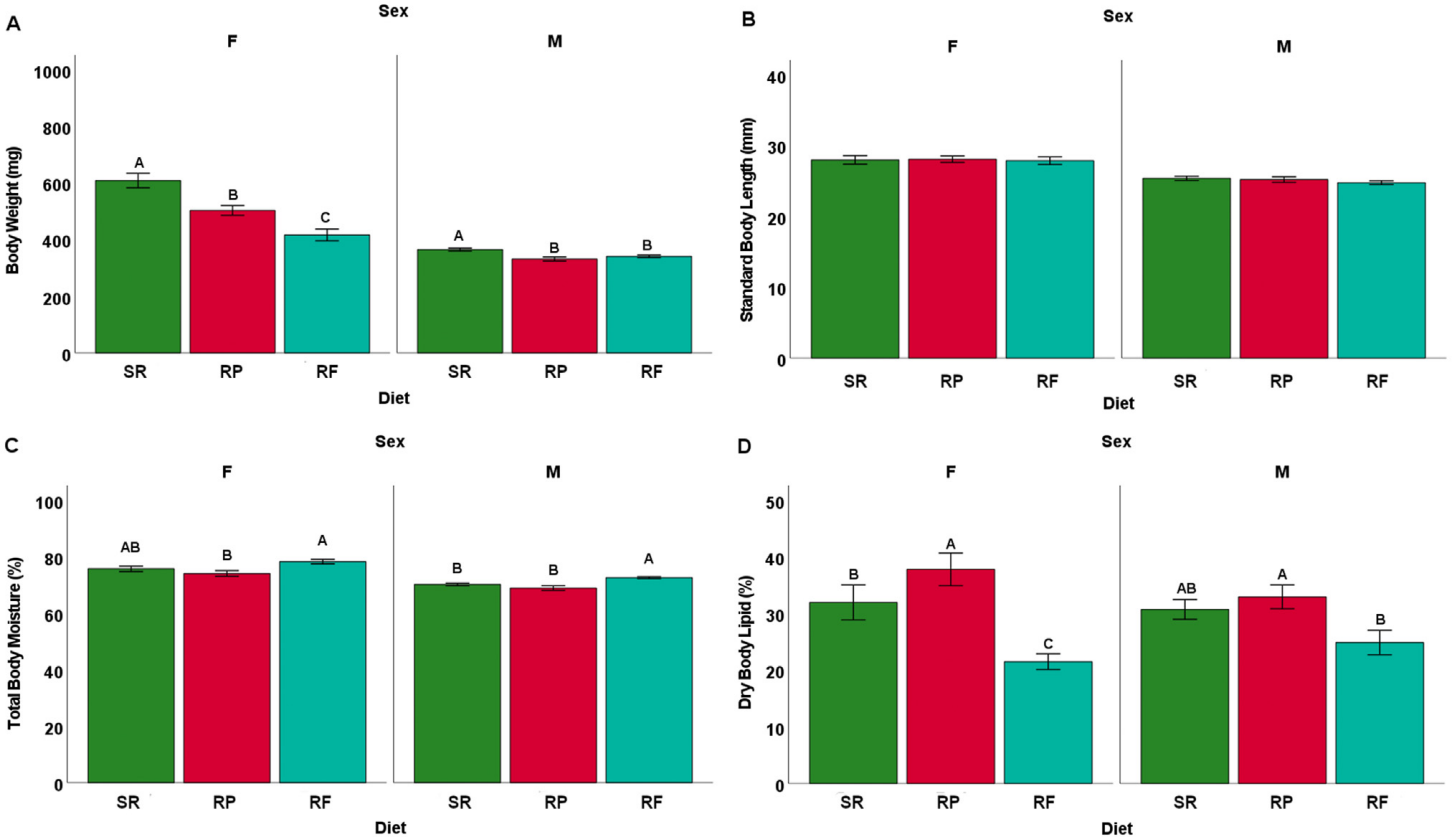
*D. rerio* body metrics at termination 16 wk on the assigned diets (*n* = 10 tanks, 14 fishes per tank for each diet treatment). The vertical bars represent the mean of body metric, and the error bars represent SEM. For each sex, different letters indicate differences among dietary treatments at *P* < 0.05, determined via an ANOVA analysis. (A) Total mean body weight for individual fish (mg) for male and female *D. rerio*. (B) Standard mean body length for individual fish (mm) for male and female *D. rerio*. (C) Total mean body moisture for individual fish (%) for male and female *D. rerio*. (D) Dry mean body lipid for individual fish (%) for male and female *D. rerio*. Sample designations are as follows: *D. rerio* fed with the standard reference diet (SR, green bars, *n* = 10 tanks), *D. rerio* fed with the reduced-protein diet (RP, red bars, *n* = 10 tanks), *D. rerio* fed with the reduced-fat (RF, blue bars, *n* = 10 tanks). The bar plot was generated via SPSS (version 26).

All 3 diets sustained *D. rerio* growth and development over the 16-wk feeding trial (Figure 1). Wet body weight of fish significantly diverged among all 3 diets over the feeding period with the largest wet body weight for *D. rerio* fed with the SR diet and the smallest wet body weight for *D. rerio* fed with the RF diet (*P* < 0.001). Terminal measures of wet body weight were separated by sex, and females had a larger wet body weight, as is typical of the species (Figure 2A). For female fish, terminal wet body weight was significantly different among all 3 diets with female *D. rerio* fed with the SR diet having the largest final body weight and female *D. rerio* fed with the RF diet having the smallest wet body weight (*P* < 0.01). For male fish, there was significantly higher wet body weight for male *D. rerio* fed with the SR diet when compared with the male *D. rerio* fed with the RF and RP diets (*P* < 0.01). Among male and female *D. rerio* fed all 3 diets, there was no difference in standard body length (Figure 2B) (*P* > 0.05). For female % body moisture, there is a significant difference between the female *D. rerio* fed with the RF diet and female *D. rerio* fed with the RP diet, with female *D. rerio* fed with the RF diet displaying higher % moisture content (Figure 2C) (*P* < 0.01). For male % body moisture, there is a significant difference between the male *D. rerio* fed with the RF diet compared with male *D. rerio* fed with the RF and RP diets, with higher moisture content in the male *D. rerio* fed with the RF diet (*P* < 0.05). For female % dry body lipid, there is a significant difference between all diet treatments. Female *D. rerio* fed with the RP diet displayed the highest percentages of body lipid content, whereas female *D. rerio* fed with the SR and RF diets displayed significantly lower percentages of body lipid (*P* < 0.05) (Figure 2D). For male % dry body lipid, significant differences are seen between male *D. rerio* with fed the RP and RF diets (*P* < 0.001).

### Breeding statistics

**FIGURE 3:**
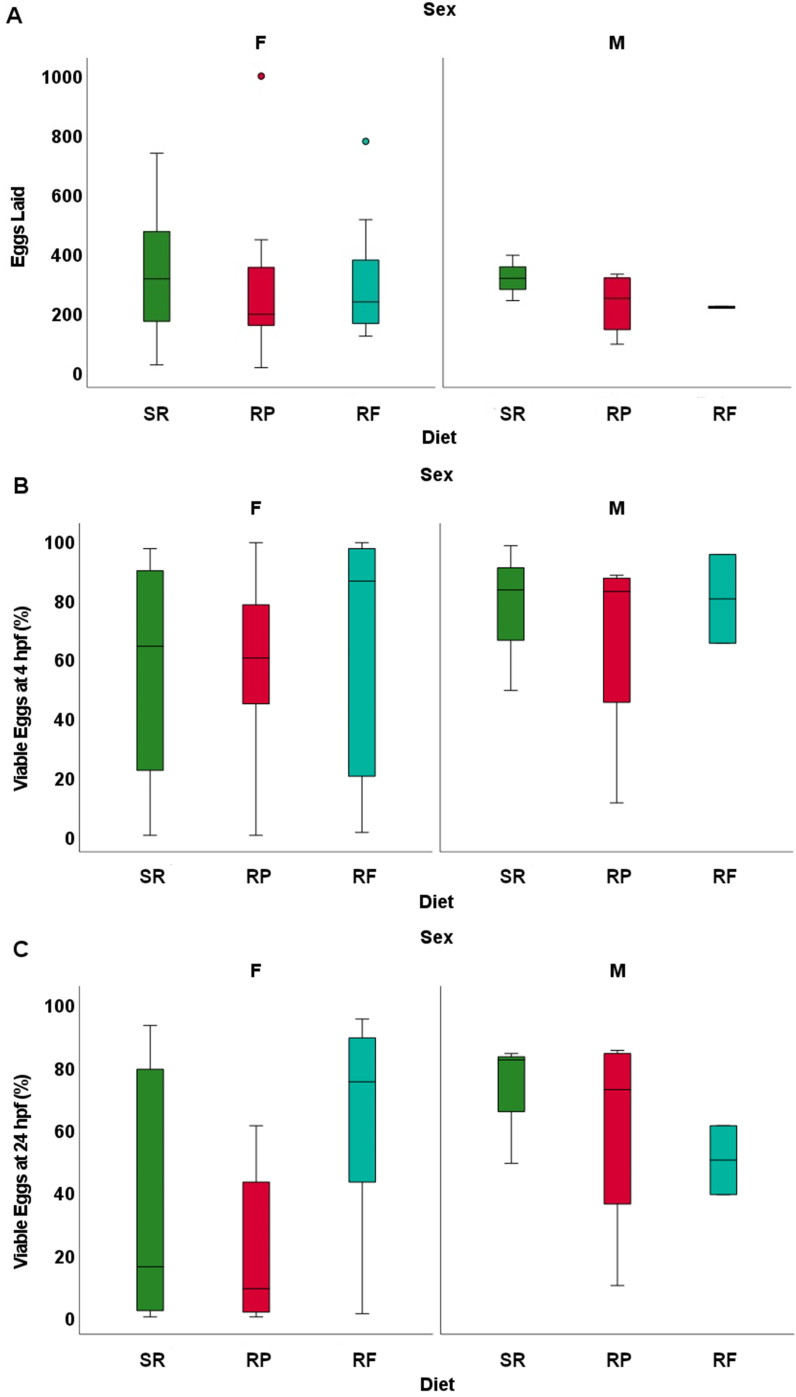
Reproductive metrics after 16–20 wk on the assigned diets for males and females outcrossed with females and males, respectively, from brook stock maintained on *Artemia* (*n* = 20 breeding events for females of the dietary treatments with a 2-wk gap, *n* = 10 breeding events for males of the dietary treatments). The vertical bars represent the mean of reproductive metrics, and the error bars represent SEM. For each sex, different letters indicate differences among dietary treatments at *P* < 0.05, determined via an ANOVA analysis. (A) Total egg produced on average for breeding pairs of male and female. (B) Egg viability (%) on average at 4 hpf for breeding pairs of male and female *D. rerio* crossed with males and females from standard diet stock. (C) Egg viability (%) on average at 24 hpf for breeding pairs of male and female *D. rerio* after 16–20 wk on the assigned diets crossed with males and females from standard diet stock. Sample designations are as follows: *D. rerio* fed with the standard reference diet (SR, green bars), *D. rerio* fed with the reduced-protein diet (RP, red bars), *D. rerio* fed with the reduced-fat (RF, blue bars). The bar plot was generated via SPSS (v.26). The RF (RF males), there was only a single instance of reproduction, and this group was not included into the analysis; however, this group is represented visually in the figure. The points outside of the box plots represent outliers.

Male and female reproduction did not differ among diet treatments for total eggs produced (*P* > 0.05), egg viability at 4 hpf (*P* > 0.05), egg viability at 24 hpf (*P* > 0.05), or successful spawning (*P* > 0.05) (not shown). For the second female spawning, there was a slightly higher proportion of viable eggs noted at 4 hpf when compared with the initial female spawning (*P* > 0.05); however, at 24 hpf, there was no significant difference between the 2 spawning events (*P* > 0.05) (Table 2, Figure 3). Male *D. rerio* fed with the RF diet only had a single observation for viability, bringing the reproducibility and real-world relevance into question and were therefore excluded from the analysis.

**TABLE 2:** Success of male and female breeding events.

Sex	Success breeding	Attempted breeding	Proportion
SR male	3	10	0.3
RP male	4	9	0.44
RF male	2	10	0.2
SR female	12	20	0.6
RP female	7	17	0.41
RF female	14	20	0.7

Attempts are pairings of males from the diet study with stock females or females from the diet study with stock males. Drops are pairings that results in eggs being released. Sample assignments are as follows: SR male = male *D. rerio* fed with the standard reference diet; SR female = female *D. rerio* female fed with the standard reference diet; RF male = male *D. rerio* fed with the reduced-fat diet; RF female = female *D. rerio* female fed with the reduced-fat diet; RP male = male *D. rerio* fed with the red diet; RP female = female *D. rerio* fed with the reduced diet.

RF, reduced-fat diet; RP, reduced-protein diet; SR, standard reference diet.

### Read quality and sample statistics

The Illumina MiSeq paired-end analysis targeting the V4 region of the 16S rRNA gene amplicons generated a raw sequence count, which resulted in 1,440,721 reads following dada2 quality checking (Supplemental Data 2). A total of 1143 observed QIIME2 features (ASVs) were identified after QIIME2 (v2022.2) analysis. The observed features and taxonomic composition are presented in the Supplemental Data 1, representing the genus level (level 6, QIIME2 v.2022.2).

### Taxonomic distribution across samples

**FIGURE 4:**
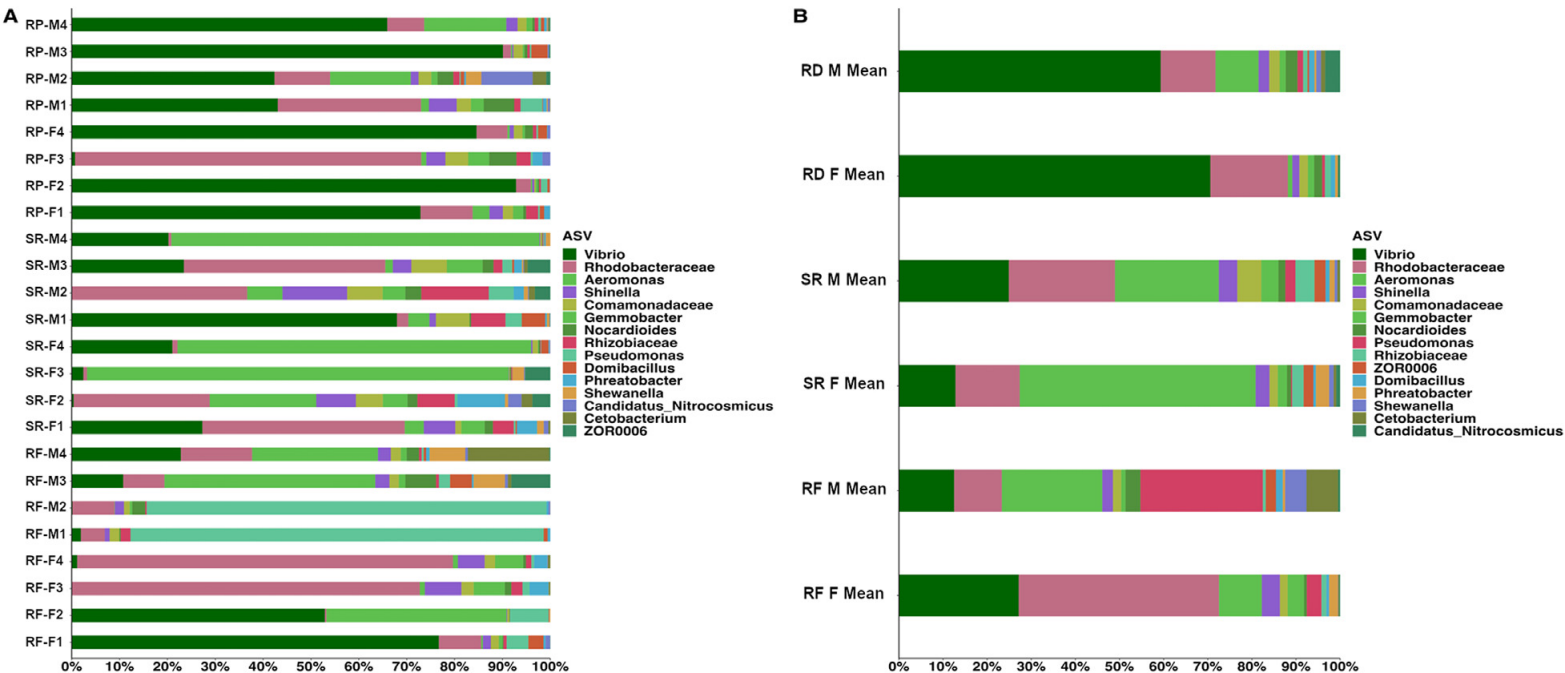
(A) Relative abundance stacked column bar graph showing top 15 taxa at the most resolved level across all samples (*n* = 4 for each sample) in the gut ecosystem of *D. rerio*. Taxonomic identities were based on their assignment through the (SILVA v138) database as determined by the Quantitative Insights into Microbial Ecology (QIIME2, v2022.2) and graphed using R (ggplot package). (B) The mean of each sample group was plotted for clarity. Sample designations are as follows: RP-M = *D. rerio* males fed with the reduced-protein diet; RP-F = *D. rerio* females fed with the reduced-protein diet; SR-M = *D. rerio* male fed with the standard reference diet; SR-F = *D. rerio* female fed with the standard reference diet; RF-M = *D. rerio* male fed with the reduced-fat diet; RF-F = *D. rerio* female fed with the reduced-fat diet. A list of taxa and their abundances is presented in Supplemental Data 1. RF, reduced-fat; RP, reduced-protein; SR, standard reference.

All samples male and female (SR, RP, and RF diet formulations) displayed Proteobacteria as the dominant phylum (TABLE 3, TABLE 4). *Vibrio*, Rhodobacteraceae, and *Aeromonas* were the primary taxon observed across all sample groups, at the highest resolution outputted via QIIME2 (2022.2) (Figure 4B, Table 5.). Female *D. rerio* fed with the SR diet were primarily composed of *Aeromonas* (∼43.9%), Rhodobacteraceae (∼14.8%), and *Vibrio* (∼11.5%). Male *D. rerio* fed with the SR diet were primarily composed of *Vibrio* (∼22.7%), *Aeromonas* (∼21.6%), and Rhodobacteraceae (∼16.7%). Female and male *D. rerio* fed with the RP diet were dominated by *Vibrio* (∼54.7% female and ∼51.6% male), and Rhodobacteraceae (∼17.9% female and ∼10.3% male). Female *D. rerio* fed with the RF diet sample group displayed high abundances of *Vibrio* (∼30.1%) and Rhodobacteraceae (∼35.8%). Male *D. rerio* fed with the RF diet were observed to display high abundances of *Pseudomonas* (∼29.9%) and *Aeromonas* (∼14.9%). A large abundance of Rhodobacteraceae (∼35.8%) was observed in the female fed with the RF diet, which was in contrast against all sample groups (Figure 4; Table 5).

**TABLE 3:** Top 10 taxa at a phylum level across male samples (*n* = 4 for each sample) in the gut ecosystem of *D. rerio*.

ASV	RF M1	RF M2	RF M3	RF M4	SR M1	SR M2	SR M3	SR M4	RP M1	RP M2	RP M3	RP M4
Proteobacteria	93.8	89	74	70.6	77.9	81.5	85.4	99.2	81.7	68.7	87.6	90.1
Fusobacteriota	0	0.1	0.6	14	0	0.9	0.7	0	0.1	2	0	0.2
Firmicutes	3.1	3.5	12.8	2.2	11.2	5.2	5.2	0.5	3.7	3	7.6	5.2
Crenarchaeota	0	0.3	0.5	0.5	0.1	0.1	0.1	0	0.3	7.7	0.2	0.1
Actinobacteriota	0.9	3.9	7.5	6.1	2.5	4.6	4	0.1	8.5	6.7	1	1.5
Planctomycetota	1.6	2.1	2.6	1.4	3.3	5.8	1.9	0.1	3.9	10.6	0.7	1
Bacteroidota	0.5	0.8	0.3	1.5	1.2	1.1	1.4	0	1	0.2	1.4	0.7
Unassigned	0	0	0.8	2	3.5	0	0.7	0	0	0	1.3	0.8
Verrucomicrobiota	0.2	0.3	0.2	0.2	0.3	0.4	0.4	0	0.8	0.9	0.1	0.2
Cyanobacteria	0	0.1	0.5	1.6	0.1	0.3	0.2	0	0	0.2	0	0.1

Taxonomic identities were based on their assignment through the (SILVA v138) database as determined by the Quantitative Insights into Microbial Ecology (QIIME2, v2022.2). The mean of each sample group was displayed for clarity. Sample assignments are as follows: SR M = male *D. rerio* fed with the standard reference diet; RF M = male *D. rerio* fed with the reduced-fat diet; RP M = male *D. rerio* fed with the reduced-protein diet. A complete list of the output of QIIME2 taxa and their abundances is presented in Supplemental Data 1.

RF, reduced-fat diet; RP, reduced-protein diet; SR, standard reference diet.

**TABLE 4:** Top 10 taxa at a phylum level across all female samples (*n* = 4 for each sample) in the gut ecosystem of *D. rerio*.

ASV	RF F1	RF F2	RF F3	RF F4	SR F1	SR F2	SR F3	SR F4	RP F1	RP F2	RP F3	RP F4
Proteobacteria	90.2	96.6	93.9	96.6	88.9	76.8	91.6	92.6	69.2	97.7	76.5	89.8
Fusobacteriota	0	0	0.3	0.5	0.4	1.7	0.1	0.1	0	0	0	0
Firmicutes	5.8	2.1	0.7	1	3.4	7.1	7.1	3.7	23	0.8	2.6	2.7
Crenarchaeota	0.8	0	0	0.1	0.8	2	0.2	0.1	0	0.1	1.2	0.5
Actinobacteriota	1.9	0.9	1.7	0.8	2.9	4.6	0.6	0.8	5.9	0.8	7.3	2.8
Planctomycetota	0.9	0.1	2.8	0.8	3.4	4.8	0.4	0.2	1.2	0.5	6.9	1.8
Bacteroidota	0.2	0.3	0.5	0.2	0.1	0.6	0	1	0.4	0	1	0.6
Unassigned	0	0	0	0	0	0	0	0.8	0	0	2.5	1.5
Verrucomicrobiota	0.2	0	0.1	0	0.2	1	0	0.6	0.2	0.1	1.7	0.2
Cyanobacteria	0	0	0	0	0	1.5	0	0.1	0	0	0.2	0.1

Taxonomic identities were based on their assignment through the (SILVA v138) database as determined by the Quantitative Insights into Microbial Ecology (QIIME2, v2022.2). The mean of each sample group was displayed for clarity. Sample assignments are as follows: RF M = female *D. rerio* fed with the reduced-fat diet; SR F = female *D. rerio* fed with the standard reference diet; RP F = female *D. rerio* fed with the reduced-protein diet. A complete list of the output of QIIME2 taxa and their abundances is presented in Supplemental Data 1.

RF, reduced-fat diet; RP, reduced-protein diet; SR, standard reference diet.

**FIGURE 5:**
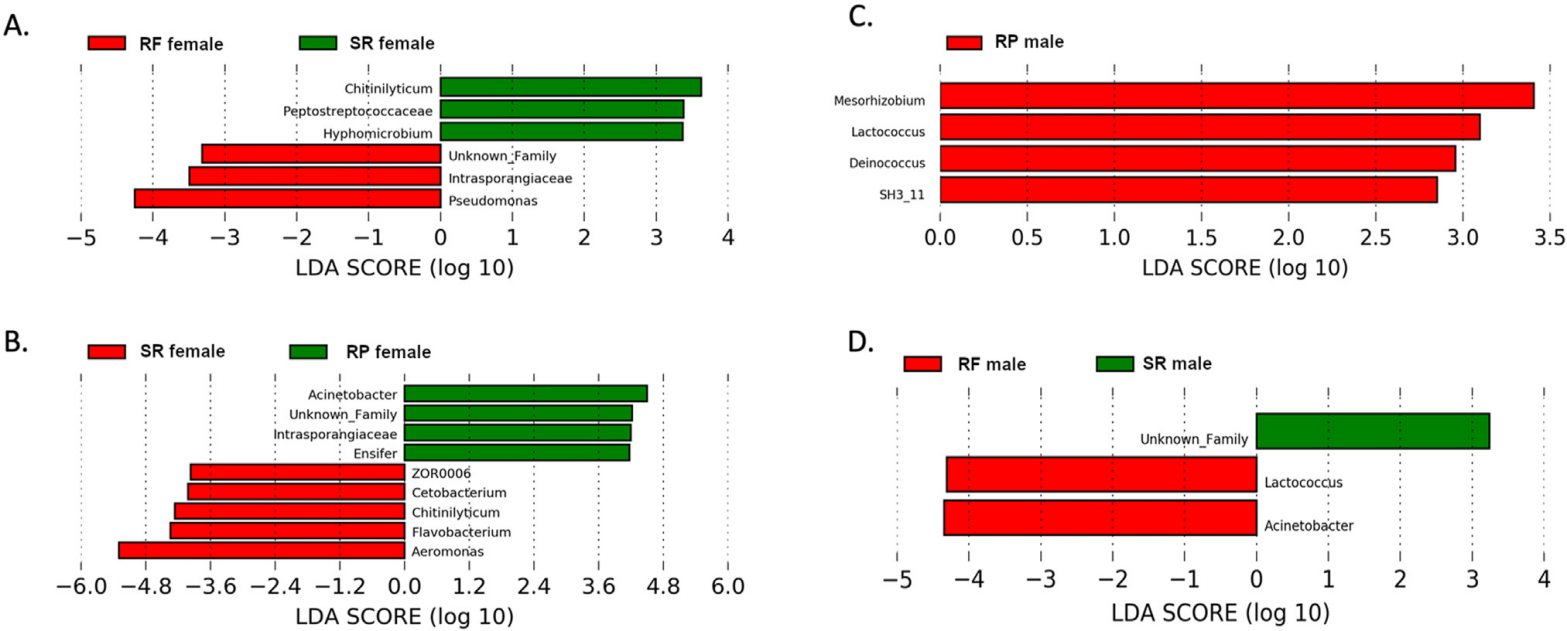
Linear discriminant analysis (LDA) effect size (LEfSe) analysis was performed on the taxonomic data of the *D. rerio* samples at the highest resolution. The effect size was visualized as a bar graph of *D. rerio* samples, (A) one class representing the female *D. rerio* fed with the standard reference diet (*n* = 4; green bars), one class representing the female *D. rerio* fed with the reduced-fat diet (*n* = 4; red bars). (B) one class representing female *D. rerio* fed with the standard reference diet (*n* = 4; red bars), one class representing female *D. rerio* fed with the reduced-protein diet (*n* = 4; green bars). (C) One class representing the male *D. rerio* fed with the reduced-protein diet (*n* = 4; red bars), this was compared with male *D. rerio* fed with the standard reference diet. (D) Male *D. rerio* fed with the standard reference diet (*n* = 4; green bars), one class representing the male *D. rerio* fed with the reduced-fat diet (*n* = 4; red bars). The values shown on the *x*-axis correspond to the log(10) effect size values at an inclusion threshold of ± 3.6.

**TABLE 5:** Top 15 taxa at the most resolved level across all samples (*n* = 4 for each sample) in the gut ecosystem of *D. rerio*.

ASV	RF female (%)	RF male (%)	SR female (%)	SR male (%)	RP female (%)	RP male (%)
Vibrio	30.075	7.35	11.45	22.725	54.675	51.6
Rhodobacteraceae	35.775	7.5	14.775	16.725	17.85	10.275
Aeromonas	9.475	14.9	43.9	21.6	0.975	7.325
Pseudomonas	3.4	29.925	0.225	2.1	0.525	1.175
Shinella	3.35	1.725	3.075	3.7	1.55	2.05
Gemmobacter	2.95	0.65	2.05	2.55	1.425	1.125
Cetobacterium	0.2	3.725	0.525	0.4	0	0.6
Rhizobiaceae	0.9	0.575	2.475	4.425	1.275	0.825
Shewanella	0.05	2.975	1	0.575	0	0.675
ZOR0006	0.025	1.775	1.975	1.65	0	0.25
Phreatobacter	1.6	0.4	2.825	0.9	0.65	0.375
Domibacillus	0.725	1.175	0.425	1	0.675	1.1
Candidatus Nitrocosmicus	0.225	0.325	0.8	0.075	0.45	2.1
Comamonadaceae	1.525	1.375	1.675	4.4	1.75	1.95
Nocardioides	0.5	2.5	0.9	1.2	1.625	2.1

Taxonomic identities were based on their assignment through the (SILVA v138) database as determined by the Quantitative Insights into Microbial Ecology (QIIME2, v2022.2). The mean of each sample group was displayed for clarity. Sample assignments are as follows: SR M mean = male *D. rerio* fed with the standard reference diet; SR F mean = female *D. rerio* fed with the standard reference diet; RF M mean = male *D. rerio* fed with the reduced-fat diet; RF M mean = female *D. rerio* fed with the reduced-fat diet; RP M mean = male *D. rerio* fed with the reduced-protein diet; RP F mean = female *D. rerio* fed with the reduced-protein diet. A complete list of the output of QIIME2 taxa and their abundances is presented in Supplemental Data 1.

RF, reduced-fat diet; RP, reduced-protein diet; SR, standard reference diet.

### Linear discriminant analysis effect size and LDA

Female *D. rerio* fed with the SR diet revealed relative abundance of *Chitinilyticum* (LDA score = 2.8), Peptostrepococcaceae (LDA score = 3.5), and *Hyphomicrobiom* (LDA score = 2.9) against female *D. rerio* fed with the RF diet*,* which revealed relative abundance of Intrasporangiaceae (LDA score = 3.5) and *Pseudomonas* (LDA score = 4.2). Male *D. rerio* fed with the SR diet revealed relative abundance of an unknown family of Gammaproteobacteria (LDA score = 3.2) against the male *D. rerio* fed with the RF diet, which revealed relative abundance of *Lactococcus* (LDA score = 4.3) and *Acinetobacter* (LDA score = 4.6). Female *D. rerio* fed with the SR diet revealed relative abundances of ZOR0006 (LDA score = 4.0), *Cetobacterium* (LDA score = 3.7), *Chitnilyticum* (LDA score = 4.3), *Flavobacterium* (LDA score = 4.3), *Aeromonas* (LDA score = 5.6), and against the female *D. rerio* fed with the RP diet, which revealed relative abundance of *Acinetobacter* (LDA score = 4.5), Intrasporangiaceae (LDA score = 4.2), and *Ensifer* (LDA score = 4.2). Male *D. rerio* fed with the SR diet revealed no distinct microbial relative abundances in contrast to the male *D. rerio* fed with the RP diet, which revealed a relative abundance of *Mesorhizobium* (LDA score = 3.7), *Lactococcus* (LDA score = 3.3), *Deinococcus*, (LDA score = 2.6), and *SH3_11* (LDA score = 3.0).

### α-Diversity and β-diversity

**FIGURE 6:**
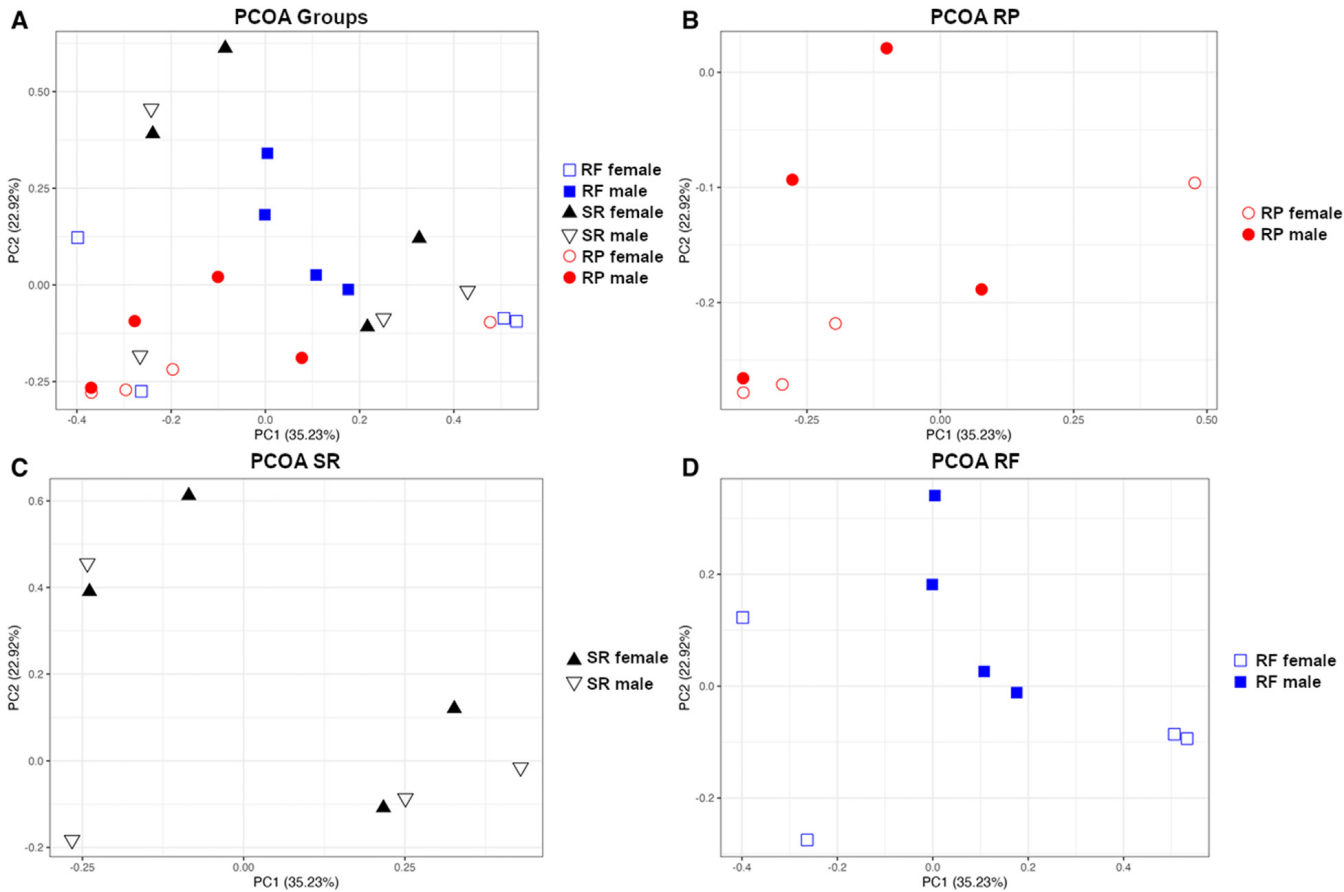
Β-Diversity analysis of gut microbiota of *D. rerio* was observed across all similarity metrics determined for the ASV table. Bray-Curtis PCOA plot to display sample clustering patterns based on observed ASVs. plotted with R (ggplot package). (A) represents all samples under one plot, and (B–D) represents samples separated for clarity. Bray–Curtis distance matrix data generated via QIIME2(v.2022.2), and the q2-qiime diversity β-group-significance. The group assignments are indicated as follows: female *D. rerio* fed with the reduced-fat diet (blue open square; *n* = 4); male *D. rerio* fed with the reduced-fat diet (blue square; *n* = 4); female *D. rerio* fed with the standard reference diet (black triangle; *n* = 4); male *D. rerio* fed with the standard reference diet (black open triangle; *n* = 4); female *D. rerio* fed with the reduced-protein diet (open red circle; *n* = 4); Male *D. rerio* fed with the reduced-protein diet (red circle; *n* = 4).

The α-diversity of the SR diet *D. rerio* samples showed insignificant taxonomic diversity as compared with the RP and RF samples (Table 6). A *t* test comparison between the alpha-diversity values of the *D. rerio* groups showed no significant (*P* > 0.05) differences using the Shannon (*P* > 0.05) and Simpson (*P* > 0.05) metrics. The microbial distribution pattern was determined utilizing Bray–Curtis metrics across all *D. rerio* samples, and then graphed in R via package ggplott. The SR diet group revealed clustering amongst the sample groups (Figure 6C). The RP diet group revealed clustering among female *D. rerio*; however, there was dissimilarity amongst the total diet group (Figure 6B). The RF diet male group revealed tight clustering among samples, and the female *D. rerio* fed with the fed RF diet revealed dissimilarity (Figure 6D). Weighted and unweighted UniFrac analyzes were conducted to account for the phylogenetic relationship (Supplemental Figure 1). The samples were clustered according to diet and sex, resulting in PERMANOVA and Adonis statistics revealing no significant dissimilarity among the groups (*R*^*2*^ = 0.262, *P* > 0.05). The samples were then clustered according to the diet, resulting in PERMANOVA (*P* < 0.05) and Adonis statistics revealing significant dissimilarity amongst the diet groups (*R*^*2*^ = 0.162, *P* < 0.05). Permdisp revealed there was a significant dispersion of samples (*P* < 0.05).

**TABLE 6:** Alpha-diversity metrics analyzed across samples (n = 4 for each sample group, separated in diet, and sex).

Sample ID	Shannon	Simpson
RF F1	3.065151929	0.68877144
RF F2	2.685135941	0.74895381
RF F3	3.05291382	0.65861197
RF F4	2.509689136	0.5470421
RF M1	3.29954611	0.83700964
RF M2	3.135436104	0.70604283
RF M3	4.402657556	0.85662666
RF M4	4.617484272	0.90733085
SR F1	4.188897849	0.86688749
SR F2	5.270400625	0.93423547
SR F3	1.709647405	0.47701708
SR F4	2.707154543	0.73085864
SR M1	4.290321511	0.84225075
SR M2	4.822240157	0.91560689
SR M3	4.129864048	0.84530501
SR M4	1.997253269	0.67907385
RP F1	4.327109122	0.84486982
RP F2	1.627899823	0.45615501
RP F3	4.448798726	0.77911513
RP F4	2.68506716	0.60261406
RP M1	4.399576495	0.87205761
RP M2	5.220783783	0.9152099
RP M3	2.481469143	0.56727863
RP M4	3.324478232	0.74779385

The α-diversity metrics were determined via qiime diversity α plugin, p-metric Shannon, and Simpson (QIIME2 v. 2022.2). Sample assignments are as follows: SR M mean = male *D. rerio* fed with the standard reference diet; SR F mean = female *D. rerio* fed with the standard reference diet; RF M mean = male *D. rerio* fed with the reduced-fat diet; RF M mean = female *D. rerio* fed with the reduced-fat diet; RP M mean = male *D. rerio* fed with the reduced-protein diet; RP F mean = female *D. rerio* fed with the reduced-protein diet.

RF, reduced-fat diet; RP, reduced-protein diet; SR, standard reference diet.

### Predicted functional analysis

**FIGURE 7:**
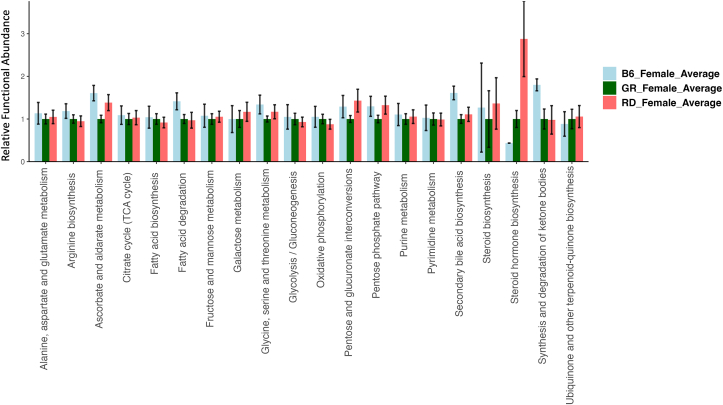
Barplot of predicted KEGG orthology (KO) metabolic functions of *D. rerio* microbiota determined through Phylogenetic Investigation of Communities by Reconstruction of Unobserved States (PICRUSt2 v2.3.0-b) script pathway_pipeline.py. light blue = female *D. rerio* fed with the reduced-fat diet (*n* = 4), dark green *n* = female *D. rerio* fed with the standard reference diet (*n* = 4), and red = female *D. rerio* fed with the reduced-protein diet (*n* = 4). The ASV table was generated from QIIME2 (v.2022.2). The *x*-axis displays the functional pathway description for each sample, and the *y*-axis displays the expression level normalized against the standard reference diet (relative functional abundance), the error bars represent the SE between sample groups. The analysis was performed using the level 2 KEGG BRITE hierarchical functional categories using PICRUSt2 (v2.3.0-b) script pathway_pipeline.py with manually curated mapfile from https://www.genome.jp/kegg-bin/get_htext?ko00001.keg (accessed on 6 September 2020).

**FIGURE 8:**
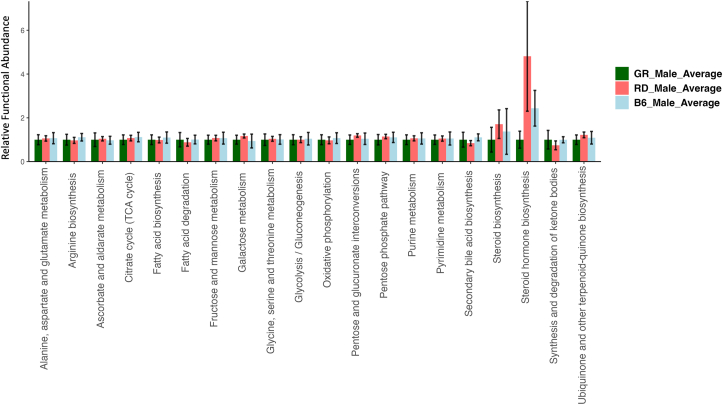
Barplot of predicted KEGG orthology (KO) metabolic functions of *D. rerio* microbiota determined through Phylogenetic Investigation of Communities by Reconstruction of Unobserved States (PICRUSt2 v2.3.0-b) script pathway_pipeline.py. Light blue = male *D. rerio* fed with the reduced-fat diet (*n* = 4), dark green *n* = male *D. rerio* fed with the standard reference diet (*n* = 4), and red = male *D. rerio* fed with the reduced-protein (*n* = 4). The ASV table was generated from QIIME2 (v.2022.2). The *x*-axis displays the functional pathway description for each sample, and the *y*-axis displays the expression level normalized against the standard reference diet (relative functional abundance), the error bars represent the SE between sample groups. The analysis was performed using the level 2 KEGG BRITE hierarchical functional categories using PICRUSt2 (v2.3.0-b) script pathway_pipeline.py with manually curated mapfile from https://www.genome.jp/kegg-bin/get_htext?ko00001.keg (accessed on 6 September 2020).

The PiCrust2 analysis revealed similarities across all sample groups; however, specific predicted pathways were upregulated/downregulated dependent on the diet received and sex of the *D. rerio* group. Female *D. rerio* fed with the RF diet revealed a minor upregulation in secondary bile acid biosynthesis (*P* > 0.05), and a significant upregulation in the synthesis and degradation of ketone bodies (*P* < 0.05). Female *D. rerio* fed with the RF diet also revealed a minor downregulation in steroid hormone biosynthesis (*P* > 0.05) (Figure 7). Male *D. rerio* fed RP diet revealed a significant upregulation in steroid hormone biosynthesis (*P* < 0.05) (Figure 8).

## Discussion

The impact of dietary protein content in our study agrees with other studies utilizing the *D. rerio* model. Peres et al. [20] reported that *D. rerio* fed a protein hydrolysate of 37.6% to 44.8% (as fed) promoted maximal weight gain and lean mass growth. In addition, Dennis-Cornelius et al. (2022), fed diets with protein content between 18% and 48% (dry weight of the diet), and the higher protein content (high protein-energy ratio) resulted in increased body weight and decreased adiposity in male and female *D. rerio*. The use of a LASSO model showed that body weight was directly impacted by dietary protein content, and body lipid content controlled for body size was inversely impacted by the dietary protein-energy ratio. In the current study, the dietary protein content (as fed) was either 47% dry matter (above the published protein requirement) or reduced to 34% dry matter of the total diet, in which protein is assumed to be limiting. In each of these studies, carbohydrates were substituted for protein in the diet when the protein content was decreased, resulting in a RP energy ratio in the lower protein content diets. Robison et al. [29] had previously concluded that dietary levels of approximately 5% and <30% did not affect growth parameters. Consequently, we conclude that dietary protein content reduced below the published protein requirement reduces lean matter production; however, the possible contribution of elevated carbohydrate in the RP leading to increased adiposity cannot be dismissed.

For dietary lipid content, results are consistent with outcomes in Fowler et al. [23], where increasing dietary lipid content in the diet increased female adiposity; however, male adiposity was impacted only by the type of dietary lipid and not the total amount. Female body weight was not impacted by total lipid level in Fowler et al. [23], but that study also fed diets with higher lipid content (8%–14% dry matter) compared with the reduced lipid diet (5.4% dry matter) in the current study and had higher total energy content (4734–5244 kcal/g diet compared with 3814 kcal/g diets in the current study). Fowler et al. [23] found no impact of total dietary lipid on early reproductive outcomes (egg production and viability), which is similar to the results of this current study. Importantly, to create a RF diet, the quantity of diatomaceous earth was increased from 12.6% to 19.7% dry matter so that all other nutrients remained constant. Although the inclusion of diatomaceous earth as a nonnutrient filler could directly affect weight gain and adiposity in combination with the reduced lipid content, this is unlikely.

We conclude that under the conditions of this study, weight gain was reduced in males and females for both the RP and the RF diets. In contrast, female adiposity increased in the RP diet but decreased in the RF diet. In males, trends were similar to females but were not statistically different. Egg production and viability were robust and not affected by the reduced nutrient diet regime. These phenotypes suggest a fundamental difference in macronutrient allocation when 1 or more macronutrients are limiting and may further depend on the class of macronutrients. Importantly, what is the role of the gut microbiome in regulating macronutrient allocation and metabolic processing when a dietary nutrient is altered?

The current analysis from the high-throughput amplicon sequencing rarefied data indicated that *Vibrio* was the most dominant taxon across all members in the gut ecosystem in *D. rerio* fed the SR, RP, or RF diets. This observation is consistent with other findings, referring to *Vibrio* as a core member of the microbiota of *D. rerio* [1,52,53]. Although studies sometimes suggest *Vibrio* members to have negative physiologic effects, *Vibrio* may potentially aid in the development of adaptive immunity [56]. The higher ionic content and anaerobic conditions in the *D. rerio* gut environment enable *Vibrio* to successfully inhabit the gut. *Vibrio* is a Gram-negative motile bacterium, in which members are glucose fermenters [57,58]. Starch, wheat or otherwise, is commonly added to aquatic feeds as an isocaloric substitution for protein and may have contributed to the increased abundance of *Vibrio* in the RP diet because *Vibrio* members have been shown previously to adhere to starch granules [59]. Rhodobacteraceae was noticeably heightened in female *D. rerio* fed the RF diet, and members of *Rhodobacteraceae* have been reported to aid in stimulating the binding of cholesterol with bile acids, and potentially inhibiting the formation of micelle formation [60,61,62]. This potentially explains the predicted upregulation of secondary bile acid biosynthesis in pathways determined via PiCrust2 KEGG pathways. This interaction potentially supports the explanation of why adiposity was significantly lower in female *D. rerio* fed with an RF diet [63]. The SR diet resulted in an abundance of the genus *Aeromonas*, which has been reported as a pathogen across marine vertebrates, and can also cause systemic illnesses in humans [64,65]. However, studies have reported beneficial members of the *Aeromonas* community. *D. rerio* mono-association with *Aeromonas veronii* was observed to increase intestinal cell proliferation in axin1 mutant *D. rerio* by upregulating Wnt signaling and β-catenin protein expression [1]. The abundance of *Aeromonas* may be concomitant with decreased abundance of *Vibrio*, potentially affected by the amount of wheat starch and diatomaceous earth in the SR diet formulation, as *Vibrio* and *Aeromonas* have been shown to compete inside the gut environment [66].

PERMANOVA statistics revealed significant differences across diets; however, PERMDISP revealed a significant dispersion among samples. The microbial communities of *D. rerio* fed the SR, RF, or RP diets displayed unique diet-specific clustering patterns of biological replicates determined via beta-diversity analysis. Notably, those male *D. rerio* fed the RF diet clustered together in the intrasample group; however, the female *D. rerio* fed with the RF diet did not show visual clustering within their intrasample group. This may be because of variation in Rhodobacteraceae and *Vibrio* among intrasample members, potentially inferring instability of microbial composition resulting from the limitation of dietary fat in the RF diet. Lipids have been reported as necessary components of *D. rerio* diets, as discussed previously [2,24]. The *D. rerio* fed with the RP diet revealed distinct clusters among females; however, male *D. rerio* fed the RP diet showed no distinct clustering among intrasample members. *D. rerio* fed the RP diet also showed similarities among microbial communities, potentially contributing to the similar distances across PC1. The variations amongst *Vibrio*, *Aeromonas*, and Rhodobacteraceae potentially contributed to the distance variations amongst the PC2, causing the dispersion on the distance matrix. Finally, *D. rerio* fed with the SR diet revealed similar distances across male and female populations. The separation among samples of the SR diet is potentially because of *Vibrio* and *Aeromonas* competitive interaction. *Vibrio* has a competitive advantage, being highly motile, whereas *Aeromonas* is primarily a nonmotile bacterium [67,68]. Typically, *Vibrio* has been shown to perturb *Aeromonas* abundances, and this observation has been related to physiologic disturbances in the gut [62].

As stated in the results, *D. rerio*-consuming diets divergent in macronutrient content did not differ significantly in survival and reproduction; however, differences were observed in growth, body composition, and microbial composition. The lack of differences in reproductive outcomes measured is not surprising, given the robust nature of early reproductive outcomes, such as gamete production and embryological viability. Long-term reproductive differences induced by dietary macronutrients have not been investigated. The PiCrust2 pathway analysis revealed an upregulation of steroid hormone biosynthesis in RP-fed male and female *D. rerio*. Body composition revealed elevated adiposity, particularly in females*,* with significant abundance of *Vibrio* compared with an SR diet. The resulting microbiome may provide a platform for a systemic inflammatory state because of the overabundance of *Vibrio* present, in which members have been linked to pathogenic and adverse effects in *D. rerio* [64]. Furthermore, an increased dry lipid mass in the RP diet may be influenced by increased steroid hormone biosynthesis, because of potential increases in cortisol or possibly C19 and C18 sex steroids. Microbial flora can synthesize, metabolize, and chemically alter steroid hormones and have been positively correlated to steroid hormones associated with energy metabolism, regulation, and immune function [69]. The specific extent to which microbes are associated with this increase in adiposity is unknown because of resolution restraints; however, this predicted upregulation requires further investigation because pathogenic bacteria are capable of influencing steroid hormone levels, which can lead to a higher susceptibility of infection [70]. The large abundance of *Vibrio* present in the RP diet microbiome could potentially influence steroid hormone ratios via host-microbe interaction, as certain *Vibrio* members have been previously shown to interact with steroid hormones [71,72]. These data indicate that an RP diet impacted adiposity, and microbial composition. Female *D. rerio* fed with an RF diet revealed a distinct upregulation of ketone body degradation and synthesis in the microbiome. We hypothesize that when dietary lipid levels are below nutritional requirements, the microbiome responds with an upregulation of ketone degradation and synthesis pathways, this hypothesis is supported via our functional predictions of the microbiome.

In conclusion, these results suggest that interactions among the diet, the gut microbial communities, associated metabolism, growth performance, and body composition in *D. rerio* are realized when specific dietary macronutrients are altered. The interactive mechanisms are unknown, but are hypothesized to be chemically mediated through a nutrient/gut/brain communication network. The changes induced via diet likely contribute to the host’s health, emphasizing the importance of feed composition and nutrient processing when *D. rerio* is used as a preclinical animal research model. A nutritionally balanced and chemically-defined diet (open formulation) should be considered as an important variable in hypothesis testing.

## Data Availability

The high-throughput amplicon sequencing datasets of *D. rerio* samples are publicly available on the BioSample Submission Portal (https://www.ncbi.nlm.nih.gov/bioproject/) under the BioProject IDs PRJNA772302 and PRJNA77230
