# Supplementary material for: Body Metrics and the Gut Microbiome in Response to Macronutrient Limitation in the Zebrafish Danio rerio
Source: Curr Dev Nutr. 2023 Mar 9;7(4):100065. doi: 10.1016/j.cdnut.2023.100065 (PMC10257228; doi:10.1016/j.cdnut.2023.100065)
Supplement: Multimedia component 3 [file mmc3.docx]

BODY METRICS AND THE GUT MICROBIOME IN RESPONSE TO MACRONUTRIENT LIMITATION IN THE ZEBRAFISH *DANIO RERIO*

George B. H. Green^1^

^1^ Department of Biology, The University of Alabama at Birmingham, 1300 University Blvd., Birmingham, AL 35294, USA


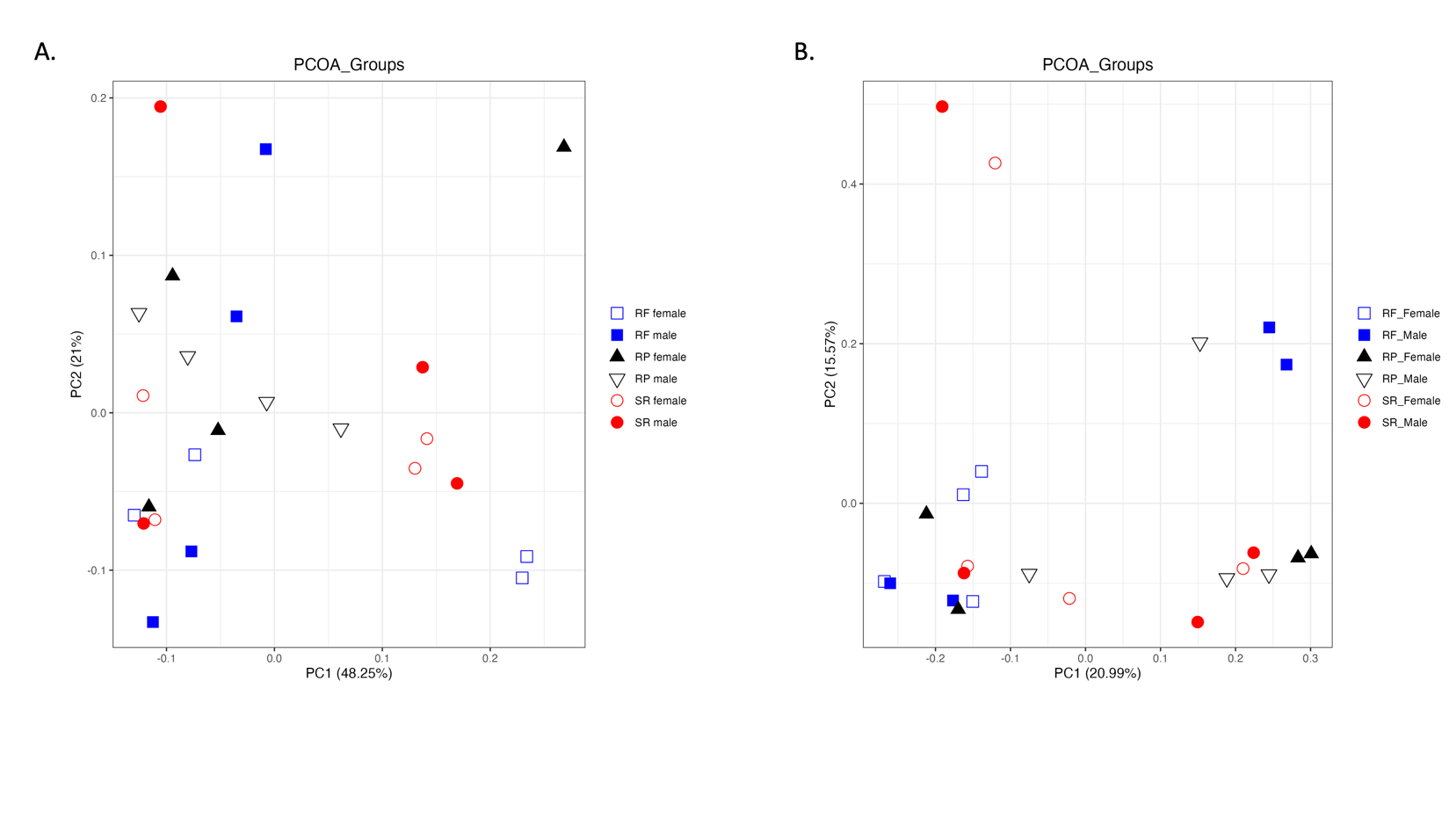


RF – Reduced Fat

SR – Standard Reference

RP – Reduced Protein

F – Female

M – Male

Supplementary Figure 1. Beta diversity analysis of gut microbiota of *D. rerio* was observed across all similarity metrics determined for the ASV table. (A.) Weighted and (B.) unweighted plot to display sample clustering patterns based on observed ASVs. plotted with R (ggplot package). Weighted and unweighted distance matrix data were generated via QIIME2(v.2022.2), and the q2-qiime diversity beta-group-significance. The group assignments are indicated as follows: female *D. rerio* fed with the reduced fat diet (blue open square; n = 4); male *D. rerio* fed with the reduced fat diet (blue square; n = 4); female *D. rerio* fed with the standard reference diet (black triangle; n = 4); male *D. rerio* fed with the Standard reference diet (black open triangle; n = 4); female *D. rerio* fed with the reduced protein diet (open red circle; n = 4); Male *D. rerio* fed with the reduced protein diet (red circle; n = 4).
